# Pathophysiology of Medication‐Related Osteonecrosis of the Jaw—A Minireview

**DOI:** 10.1002/jbm4.10785

**Published:** 2023-06-22

**Authors:** Sotirios Tetradis, Matthew R. Allen, Salvatore L. Ruggiero

**Affiliations:** ^1^ Division of Diagnostic and Surgical Sciences UCLA School of Dentistry Los Angeles CA USA; ^2^ Department of Anatomy, Cell Biology & Physiology Indiana University School of Medicine Indianapolis IN USA; ^3^ New York Center for Orthognathic and Maxillofacial Surgery Lake Success NY USA; ^4^ Department Oral and Maxillofacial Surgery Stony Brook School of Dental Medicine Stony Brook NY USA; ^5^ Division of Oral and Maxillofacial Surgery Hofstra‐Northwell School of Medicine Hempstead NY USA

**Keywords:** DISEASES AND DISORDERS OF/RELATED TO BONE, ANIMAL MODELS, PRECLINICAL STUDIES, BONE MODELING AND REMODELING, MOLECULAR PATHWAYS‐REMODELING

## Abstract

Medication‐related osteonecrosis of the jaw (MRONJ) is a rare but serious adverse effect of antiresorptive medications administered for control of osseous malignancy, osteoporosis, or other bone metabolic diseases. Despite being reported in the literature two decades ago, MRONJ etiology, pathophysiology, and progression remain largely unknown, and current nonoperative or operative treatment strategies are mostly empirical. Several hypotheses that attempt to explain the mechanisms of MRONJ pathogenesis have been proposed. However, none of these hypotheses alone is able to capture the complex mechanistic underpinnings of the disease. In this minireview, we aim to highlight key findings from clinical and translational studies and propose a unifying model for the pathogenesis and progression of MRONJ. We also identify aspects of the disease process that require further investigation and suggest areas for future research efforts toward calibrating methodologic approaches and validating experimental findings. © 2023 The Authors. *JBMR Plus* published by Wiley Periodicals LLC on behalf of American Society for Bone and Mineral Research.

## Graphical abstract

The etiology of medication‐related osteonecrosis of the jaw (MRONJ), a rare but serious adverse effect of antiresorptive medications, remains largely elusive. This minireview highlights key findings from clinical and translational studies and proposes a unifying model for the pathogenesis and progression of MRONJ.

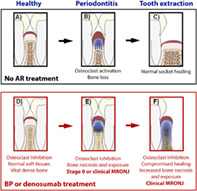

## Introduction

Medication‐related osteonecrosis of the jaw (MRONJ) is defined as exposed bone or bone that can be probed through a fistula in the maxillofacial region that has persisted for more than 8 weeks in patients with current or previous treatment with antiresorptives alone or in combination with immune modulators or antiangiogenic medications in the absence of prior radiation therapy or metastatic jaw disease.^(^ ^1^, ^2^ ^)^ Current treatments of MRONJ are mostly empirical and, depending on the severity of the disease, range from observation, antibiotics, and oral hygiene to sequestration and segmental resection of the affected necrotic bone.^(^ ^2^ ^)^

Medications that have been associated with MRONJ include bisphosphonates (BPs), denosumab, and romosozumab.^(^ ^2^ ^)^ BPs exert their effect by binding to the bone mineral and upon their release during active remodeling are internalized by osteoclasts and inhibit osteoclast function by blocking significant metabolic pathways and inducing cell apoptosis.^(^ ^3^ ^)^ Denosumab binds to and inhibits the receptor activator of NF‐κB ligand (RANKL). In bone, RANKL is produced by several cell types, including osteoblasts, osteocytes, stromal bone cells, and immune cells and is a major regulator of osteoclast precursor commitment and osteoclast differentiation and function.^(^ ^4^ ^)^ Through its RANKL binding action, denosumab inhibits osteoclastic numbers and activity.^(^ ^5^ ^)^ Romosozumab binds to and inhibits sclerostin, a molecule predominantly expressed in osteocytes that is an inhibitor of Wnt signaling. By inhibition of sclerostin, romosozumab activates Wnt signaling in osteoblastic precursor cells, mature osteoblasts, and osteocytes and induces bone formation, while inhibiting bone resorption. Romosozumab has recently entered the medical field and, although its pharmacologic action on sclerostin inhibition is well understood, the biologic mechanisms of its anabolic and anticatabolic effects are not well delineated.^(^ ^6^ ^)^

Local risk factors associated with the development of MRONJ have been identified.^(^ ^2^ ^)^ These include surgical procedures such as tooth extraction or implant placement, the presence of dental disease such as periodontal or periapical disease, and mechanical trauma through ill‐fitting dentures or mastication in anatomically prone areas.^(^ ^7^ ^)^ Tooth extraction is the most prevalent factor predisposing to MRONJ and reported present in 60% to 80% of cases.^(^ ^8^, ^9^, ^10^ ^)^ A small percentage of cases do not appear to be associated with any obvious local factors and are characterized as spontaneous MRONJ.^(^ ^11^ ^)^ Whether these cases are truly spontaneous or the result of an unidentified factor that predisposes to the disease development remains unknown, although mostly likely the latter.

Although MRONJ was initially reported in 2003^(^ ^12^, ^13^ ^)^ and described in 2004,^(^ ^14^ ^)^ the pathophysiology of the disease remains largely unknown. Multiple hypotheses have been proposed to explain the mechanisms of MRONJ development.^(^ ^15^, ^16^, ^17^, ^18^, ^19^, ^20^, ^21^, ^22^, ^23^, ^24^, ^25^, ^26^, ^27^ ^)^ These hypotheses include but are not limited to:

- an increased accumulation of bisphosphonates in the jaws due to a high rates of bone turnover;
- a direct toxic effect of bisphosphonates in the oral mucosa;
- a higher sensitivity of oral cell types, such as osteoblasts, periodontal fibroblasts, or mesenchymal stem cells to bisphosphonates;
- a distinct sensitivity of the jaws versus long bones to developing osteonecrosis reflected by the diverse composition, embryologic origin (neural crest versus mesoderm), or developmental pattern (intramembranous versus endochondral);
- an altered immune response of the oral tissues to injury or infection;
- defective wound healing of the oral tissues in the presence of antiresorptives;
- inhibition of angiogenesis;
- a genetic framework that predisposes patients to MRONJ development;
- systemic comorbidities that compound the healing of the oral tissues.

However, none of these theories alone can fully explain the disease process and capture all disease attributes. It appears that MRONJ is a multifactorial disease that involves multiple events that occur simultaneously and create the conditions that precipitate the development of the disease.

## Key Parameters for Formulating a Model for MRONJ Pathophysiology

The purpose of this brief review is not to review the different hypotheses and provide supporting evidence for each one of them. This has been done, in detail, in many prior publications (see above). Moreover, our goal is not to perform an exhaustive review of the literature and report on all significant findings of detailed processes, target molecules, or cell types that might participate in the disease process. Rather, our goal is to provide a unique angle by highlighting published evidence from clinical and translational studies and propose a unifying model for the pathogenesis and progression of the disease. We also aim to identify areas of the disease process that remain unknown and provide recommendations for areas of future research efforts toward calibrating methodologic approaches, enhancing validity of experimental findings, and uncovering mechanistic understanding of the disease for improved therapeutic interventions.

In formulating a model that outlines the process of MRONJ onset, establishment, and progression, we have considered findings from clinical, translational, and in vitro studies combined with our clinical experiences from MRONJ patient care and our understanding of experimental models of disease, their advantages, disadvantages, and technical challenges, as well as the principles of basic bone homeostasis. Key parameters that we have integrated in this model include:

- Consideration of only BPs and denosumab and their association with MRONJ. Robust clinical and translational observations provide strong evidence of the association between antiresorptives (BPs and denosumab) with MRONJ.^(^ ^1^, ^2^ ^)^ Although romosozumab treatment has been linked to a low risk of MRONJ, only a few MRONJ cases have been reported, without any clinical or radiographic findings and treatment outcomes.^(^ ^28^, ^29^ ^)^ Given the limited information on romosozumab and MRONJ, additional research is needed to refine its association and risk estimate for MRONJ. Similarly, MRONJ‐like lesions have been reported in patients not on antiresorptives, but treated with antiangiogenics, immunomodulators, chemotherapeutics, or tyrosine kinase signaling inhibitors.^(^ ^30^, ^31^, ^32^ ^)^ The evidence linking such medications to MRONJ is not strong.^(^ ^2^ ^)^ Furthermore, whether the pathophysiologic mechanisms of bone exposure associated with antiresorptives versus other medications is the same is unknown. As such, we have excluded romosozumab and other medications from the proposed model.
- The central role of inhibited osteoclastic activity as disease instigator. This is strongly supported by the fact that two pharmacologic agents (bisphosphonates and denosumab) that potently target/inhibit osteoclast function through diverse mechanisms result in indistinguishable clinical and experimental disease phenotypes. The only difference between bisphosphonates and denosumab that has been reported is the higher incidence of MRONJ in osteoporotic denosumab users.^(^ ^2^ ^)^ But even this difference could be likely explained by the more robust resorption inhibition effected by denosumab.
- Nonosteoclastic effects caused by bisphosphonates as etiologic factors in MRONJ pathogenesis without supporting experimental or at least theoretical evidence that denosumab could cause similar effects or vice versa. This would include the great majority of in vitro reports on direct effects of bisphosphonates in various cell populations, including osteoblasts, fibroblasts, endothelial cells, periodontal ligament cells, keratinocytes, stem cells, etc.^(^ ^33^ ^)^
- The soft tissue deterioration as a result of the disease process and not as a direct effect of antiresorptives. Although there is evidence that bisphosphonates are detected in saliva,^(^ ^34^ ^)^ and although direct effects of bisphosphonates in the gastric mucosa in vivo and in oral mucosal cells in vitro are well described,^(^ ^35^, ^36^, ^37^ ^)^ evidence of bisphosphonates affecting the oral mucosa in vivo or of denosumab being secreted in the saliva or having a direct effect on oral mucosa cells are lacking. Thus, soft tissue deterioration during the MRONJ process is most likely the result of the disease progression and not a direct toxic effect of the antiresorptive medications.
- Bone necrosis as an initiating event during MRONJ onset. Strong clinical evidence has established the existence of stage 0 MRONJ, a phase of the disease in which no exposed bone is observed clinically, but the patient presents with nonspecific symptoms, clinical findings not explained by common dental disease or radiographic findings such as altered trabecular architecture, bone sclerosis, lack of osseous socket healing, sequestration, or periosteal bone formation.^(^ ^2^, ^38^, ^39^, ^40^, ^41^ ^)^ Importantly, 50% of stage 0 MRONJ cases transition to stage 1 or 2 MRONJ with clinical bone exposure within 6 months of original diagnosis.^(^ ^42^ ^)^ Evidence from translational studies has shown that although bone necrosis is present in nearly all animals receiving antiresorptives and experimental intervention, bone exposure is found in a fraction of these animals with bone necrosis.^(^ ^43^, ^44^, ^45^, ^46^, ^47^, ^48^ ^)^ These clinical and translational observations combined with the lack of in vivo evidence of a direct effect of antiresorptives in the oral mucosa strongly support bone necrosis as an early event in MRONJ onset, with soft tissue breakdown after bone necrosis or being the direct effect of traumatic intervention, such as tooth extraction or implant placement.

## Proposed Clinical Model of MRONJ Pathophysiology

In constructing a model for MRONJ pathophysiology, first we considered the healthy equilibrium of the oral cavity, particularly of the oral mucosa, teeth, and underlying alveolar bone, and how this equilibrium is altered at the setting of dental disease or oral trauma. Then we considered how this balanced equilibrium in health and unbalanced equilibrium in the presence of dental disease or oral trauma would be affected by the inhibition of osteoclastic activity in the presence of antiresorptive treatment.

### Jaw homeostasis in the absence of antiresorptives

#### Healthy equilibrium

The maxilla and mandible are covered by masticatory mucosa with keratinized or parakeratinized stratified squamous epithelium in the attached gingiva and hard palate or by lining mucosa with nonkeratinized stratified squamous epithelium in the areas of free gingiva and vestibule. The oral epithelium is attached to the noncellular basement membrane, which connects it to the lamina propria, a thin layer of loose connective tissue, and the submucosa, a dense layer of fibrocollagenous and elastic connective tissue. The submucosa is absent in the areas of attached gingiva and hard palate covered by masticatory mucosa, and the lamina propria is directly bound to the jaw bone.^(^ ^49^, ^50^ ^)^ Fibroblasts, macrophages, mast cells, and sparse inflammatory cells can be found in the lamina propria, in addition to blood vessels, nerves, and fibers immersed in an amorphous substance composed of proteoglycans and glycoproteins.^(^ ^51^ ^)^ A healthy oral mucosa, with the continued proliferation and exfoliation of epithelium and the presence of specialized immune cells, provides a barrier against most microorganisms and inhibits their penetration into deeper tissues.^(^ ^52^ ^)^

The maxilla and mandible demonstrate typical osseous architecture. The maxilla shows thin cortical outlines and sparse trabeculation, particularly in the posterior sextants, whereas the mandible demonstrates thick cortical outlines and dense trabeculation. Both bones are covered by a well‐defined periosteal layer, except in areas of muscle attachment or the temporomandibular joint.^(^ ^49^ ^)^ However, the jaws have specialized functions and display distinct responses to developmental, mechanical, and homeostatic stimuli.^(^ ^53^ ^)^ Developmentally, the jaws originate from the neural crest (the ectomesenchyme) and not from the mesoderm,^(^ ^54^ ^)^ and undergo intramembranous versus endochondral ossification.^(^ ^55^ ^)^ Growth factors and signaling pathways can have distinct roles in the craniofacial versus axial and appendicular skeleton.^(^ ^56^, ^57^ ^)^ Osteoblasts and osteoclasts from the jaws show diverse differentiation potential versus their counterparts from other skeletal sites.^(^ ^58^, ^59^, ^60^, ^61^ ^)^ Single‐cell analysis of mandibular versus long‐bone marrow reveals skeletal site‐specific differences in the immune microenvironment of these two bones.^(^ ^62^, ^63^ ^)^ The teeth are held in the sockets through the periodontal ligament, containing a dense network of Sharpey's fibers extending from the root cementum to a thin layer of bundle bone, called lamina dura, that lines the wall of the tooth socket.^(^ ^49^ ^)^

**Fig. 1:**
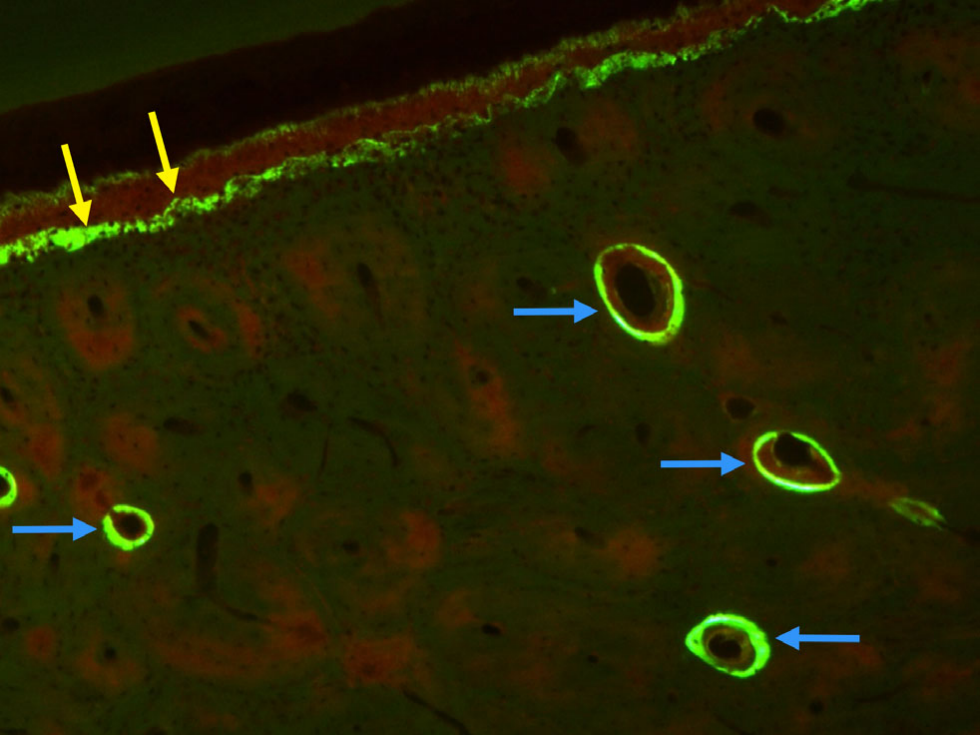
Bone remodeling occurs both within cortical bone and on bone surfaces. A central tenet of bone physiology is remodeling, where the coordinated spatial and temporal actions of osteoblasts and osteoclasts renew damaged/compromised bone tissue. Remodeling occurs both within cortical bone (intracortical/osteonal remodeling) and on bone surfaces. Image taken from a dog mandible (~15 months old). Blue arrows point to osteonal remodeling within the alveolar cortical bone. Yellow arrows point to surface remodeling on the surface adjacent to the tooth.

Under baseline conditions, bones of the jaw undergo both modeling and remodeling.^(^ ^64^, ^65^, ^66^ ^)^ Modeling, the process through which bone at a given location is either resorbed or formed, occurs mainly during development and growth of the jaws. During modeling, various surfaces of the bone either form bone through osteoblast activity or resorb bone through osteoclast activity. Remodeling is the process where a given area undergoes osteoclast resorption followed by osteoblast formation at the same spatial area.^(^ ^67^ ^)^ Bone remodeling occurs on bone surfaces, and intracortically (Fig. 1).^(^ ^68^ ^)^ This intracortical remodeling process is essential for maintaining the integrity of the bone, replacing regions of bone with microdamage, nonviable osteocytes, or areas with abnormal mineralization/organic matrix. Most bones have capacity to both model and remodel. One unique property of jaw remodeling is that there are regions with very high remodeling rate. For example, the alveolar bone has one of the highest rates of intracortical remodeling in the whole skeleton, whereas the basal region demonstrates very low remodeling rate.^(^ ^69^ ^)^

In the absence of any local or systemic disease, the soft, osseous and dental tissues of the oral cavity respond to physiologic functional stimuli of mastication and demonstrate a normal appearance.

#### Local disease/local trauma

Several pathologies affect the oral environment and alter jawbone homeostasis. The most common pathologic process of the jawbones involve extension of infectious dental disease to the alveolar bone through the gingivae (periodontitis) or through the tooth pulp (periapical disease).^(^ ^70^, ^71^ ^)^ In addition, localized trauma, including tooth extraction, implant placement, ill‐fitting dentures, or increased masticatory forces at sites such as tori, exostosis, or the lingual mandibular cortex at the retromolar area, can affect the soft and hard tissues of the oral cavity.^(^ ^72^, ^73^ ^)^

**Fig. 2:**
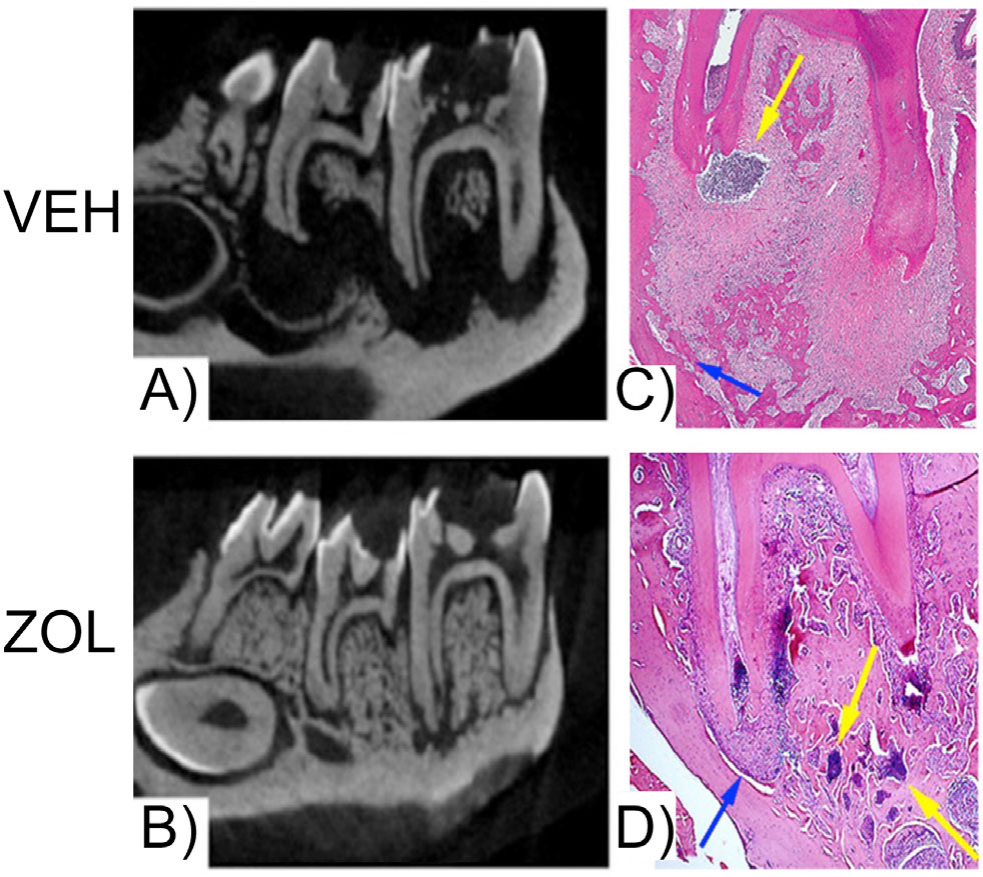
Response of alveolar bone to periapical disease in vehicle (Veh)‐ and zoledronate (ZOL)‐treated animals. (*A*) μCT sections demonstrate significant periapical bone loss in Veh‐treated animals. (*B*) Inhibition of bone resorption by ZOL results in minimal periapical bone loss. (*C*) Histologic assessment of Veh‐treated animals demonstrates bone resorption away from the nidus of the infection at the root apex. A strong inflammatory response with abscess formation at the apical areas and a more fibrous periphery are noted. (*D*) In ZOL‐treated animals, the bone is in very close proximity to the apex. A strong inflammatory response at the nidus of the infection at the root apex is noted. The inflammatory infiltrate extends to the marrow spaces of the periapical bone. Blue arrows point to the extent of periapical bone loss. Yellow arrows point to areas of inflammatory infiltrate. (Figure modified from Hadaya and colleagues^(^ ^90^ ^)^).

Dental disease that extends in the periapical or periodontal tissues causes localized infection, inflammation, and associated bone loss.^(^ ^70^, ^71^ ^)^ This physiologic response of the oral tissues localizes the infection in the peri‐radicular tissues and limits its spread. The bone resorbs away from the infection nidus, creating space for mounting of an effective inflammatory response and protecting the bone from the toxic inflammatory environment (Fig. 2). Indeed, in periodontal and periapical disease, a microbial biofilm forms on the root or interradicular surface and is surrounded by an intense inflammatory infiltrate. At the periphery of the lesion, activated osteoclasts are observed along the resorbing alveolar bone.^(^ ^70^, ^74^, ^75^, ^76^ ^)^ In periodontitis, increased apoptosis of inflammatory cells, periodontal cells, and osteocytes is observed. Osteocytes undergoing apoptosis secrete cytokines and particularly RANKL that stimulates osteoclast formation and activity and results in the resorption of the dying bone. The continued presence of periodontal or periapical disease results in further destruction of periodontal tissues and associated bone loss.^(^ ^77^ ^)^ In periodontitis, the biofilm formation on the tooth surface and resultant inflammatory changes alter the junctional epithelium at the dento‐gingival junction and induce loss of cellular continuity and detachment from the tooth surface and conversion to pocket epithelium. The residual junctional epithelium migrates apically and the periodontal pocket deepens.^(^ ^70^ ^)^ The above changes lead to decreased tooth support and eventually tooth mobility and tooth loss. Intervention to reverse the disease progression through plaque removal, endodontic treatment, or tooth extraction aim to eliminate the infection, resolve the inflammation, and induce healing of the soft and hard alveolar tissues.

Similar to dental disease, localized trauma follows an analogous process. The oral cavity is covered by keratinized or nonkeratinized epithelium and a thin mucosal lining.^(^ ^49^ ^)^ The oral mucosa is particularly thin in areas of bone exostoses (torus palatinus, torus mandibularis, buccal exostoses) or at edentulous alveolar ridge sites with a knife‐edge ridge shape. As such, injury to the oral mucosa can be in close proximity to the maxillary or mandibular bone. In addition, iatrogenic interventions, such as tooth extractions or implant placement, induce trauma to the oral cavity that extends and includes the jawbone. A healthy response of the bone to this physiologic, paraphysiologic, or iatrogenic injury engages multiple cell types with the osteoclasts playing a key role in bone remodeling and normal healing.^(^ ^72^, ^73^ ^)^ In periodontal disease, increased osteocyte apoptosis is observed.^(^ ^46^, ^77^ ^)^ Similarly, in localized trauma, the bone resorbs away from the site of increased forces, while the oral mucosa can develop a traumatic ulcer if the mechanical injury persists.^(^ ^78^, ^79^ ^)^ During tooth extraction, bone necrosis ensues at early time points.^(^ ^43^, ^80^ ^)^ The inflammatory response caused by the injury induces formation and activation of osteoclasts that contribute to the remodeling of the original socket outline and removal of the necrotic bone at the periphery of the socket after the extraction.^(^ ^72^ ^)^ This osteoclastic activity is accompanied by a strong differentiation of periodontal and marrow mesenchymal stem cells to osteoblasts that form woven bone and fill in the socket from its periphery.^(^ ^81^, ^82^ ^)^ Subsequent activity removes a large amount of the woven bone and transitions the remaining bone into normal lamellar structured trabeculae and cortex.^(^ ^72^ ^)^ Finally, during implant placement, the coordinated function of recruited osteoclasts and differentiated osteoblasts at the implant site create a close association of the bone with the implant surface and successful osseointegration of the implant fixture.^(^ ^73^, ^82^ ^)^

### Jaw homeostasis in the presence of antiresorptives

#### Healthy equilibrium

Homeostasis and remodeling of alveolar tissues is fundamentally altered during osteoclast inhibition. In the absence of dental disease, reduced osteoclast function leads to suppression of bone remodeling^(^ ^69^ ^)^ and an overall increased bone mass,^(^ ^83^ ^)^ similar to other skeletal sites. Radiographically, this presents as an overall increase in radiographic bone density and increased thickness of cortical outlines and lamina dura around the tooth socket outline.^(^ ^84^, ^85^ ^)^ No mucosal or submucosal changes are observed.^(^ ^45^, ^46^, ^47^ ^)^ During the physiologic function of the oral tissues, these osseous changes are well tolerated and do not generate clinical, radiographic, or histologic signs of MRONJ.

#### Periodontal or periapical disease or local trauma

The progression of periodontal or periapical disease are severely compromised during inhibition of resorption. At the very early disease stages of periodontal or periapical disease, when only soft tissues are involved, it is reasonable to assume that no differences between the absence or presence of antiresorptives would be observed. However, as the infection persists and inflammatory changes expand to involve the alveolar bone, the defective osteoclastic function and/or reduced osteoclast numbers compromise the ability to remove the alveolar bone away from the inflammatory nidus (Fig. 2).^(^ ^45^, ^46^, ^47^, ^86^, ^87^, ^88^, ^89^, ^90^ ^)^ Similar observations are found in local trauma, where the absence of osteoclastic activity inhibits remodeling of original bone during palatal wounding,^(^ ^80^, ^91^ ^)^ or tooth extraction.^(^ ^90^, ^92^, ^93^, ^94^ ^)^

**Fig. 3:**
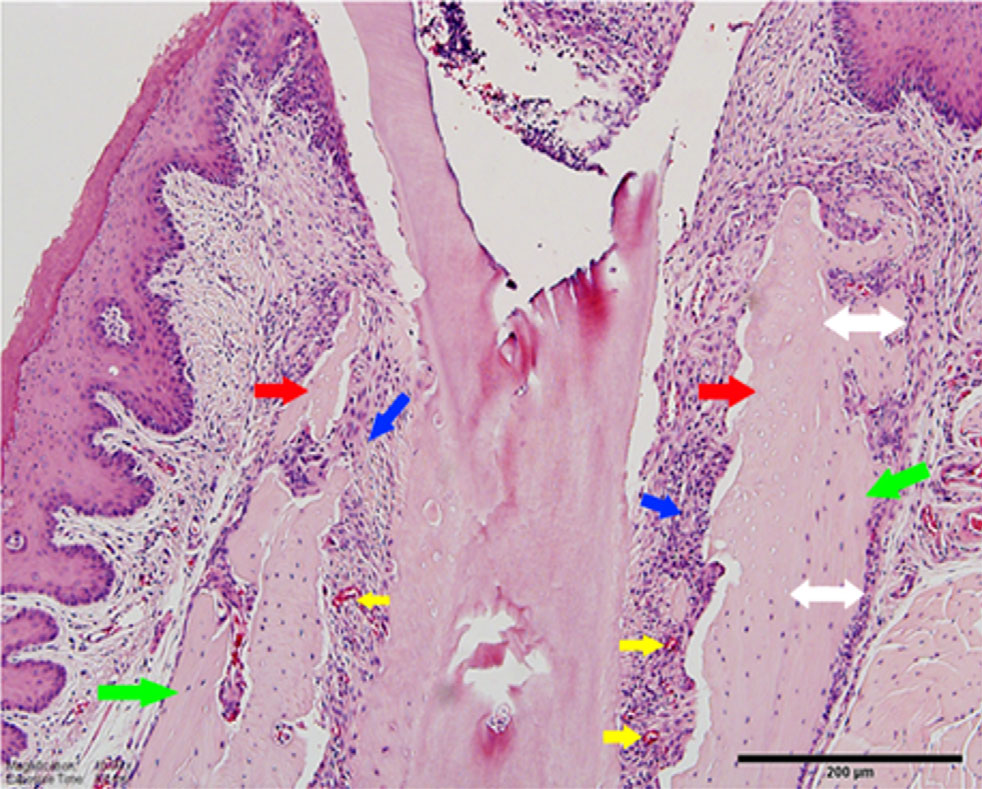
Onset of medication‐related osteonecrosis of the jaw (MRONJ) around teeth with periapical disease in zoledronate (ZOL)‐treated mice. Red arrows point to localized osteonecrotic areas at the alveolar crest marked by empty osteocytic lacunae, green arrows to presence of osteocytes marking vital bone, double white arrows to periosteal bone deposition adjacent to osteonecrotic areas, blue arrows to inflammatory infiltrate, and yellow arrows to vascular structures. (Figure modified from Kang and colleagues^(^ ^46^ ^)^).

The inflammatory environment is toxic for osteocytes. Indeed, inflammatory cytokines, such as TNF‐a, IL‐6, and IL‐1, can induce osteocyte apoptosis. Apoptosing osteocytes secrete signals that either directly or indirectly activate osteoclasts that resorb the necrotic bone.^(^ ^77^ ^)^ However, in the presence of antiresorptives and inhibition of osteoclastic function, the necrotic bone is retained.^(^ ^45^, ^46^, ^47^, ^48^, ^88^, ^89^, ^90^, ^95^ ^)^ Empty osteocytic lacunae accumulate in sites adjacent to inflammation and not throughout the alveolar bone, supporting a local niche that favors osteonecrosis rather than a generalized and uniformly distributed bone necrosis.^(^ ^46^, ^96^ ^)^ In addition, the rate of osteocyte death during MRONJ establishment and progression is enhanced and the extent of necrotic bone increases (Fig. 3).^(^ ^46^ ^)^ During local trauma by palatal denudation, a considerably more extensive osteonecrosis is observed in the presence of zoledronate (ZOL).^(^ ^80^ ^)^

Osteocytic death during MRONJ likely involves multiple mechanisms, including apoptosis and cell necrosis, and is observed at early stages of disease setting.^(^ ^97^, ^98^, ^99^ ^)^ Dying osteocytes release cellular signals that enhance the degree and severity of periodontal tissue inflammation. The presence of necrotic bone that cannot be removed further inhibits healing of periodontal tissues. Changes in the oral submucosa adjacent to developing areas of osteonecrosis are observed as early as 1 week and before bone exposure. Such changes include a decreased arteriole and venule network and increased expression of oxidative stress, hypoxia, and cell apoptosis markers.^(^ ^100^ ^)^ Furthermore, the network of collagen fibers connecting the submucosa to the alveolar bone is interrupted in areas of osteonecrosis.^(^ ^94^ ^)^

As a result, an overall increase in the inflammatory infiltrate of the periodontal tissues adjacent to the necrotic bone and an altered inflammatory response are noted.^(^ ^46^, ^47^, ^48^, ^87^, ^90^, ^101^, ^102^ ^)^ Similarly, in local trauma by palatal wounding or tooth extraction, an intense neutrophil aggregation is observed adjacent to the necrotic bone in the presence, but not in the absence, of antiresorptives.^(^ ^103^, ^104^ ^)^ Importantly, during MRONJ development, there is a shift toward a prolonged pro‐inflammatory environment.^(^ ^15^, ^19^ ^)^ Suppression of adaptive regulatory T cells (Tregs) and activation of inflammatory T‐helper‐producing interleukin 17 cells (Th17) are observed.^(^ ^44^ ^)^ Prolonged duration of ligature‐induced periodontitis increases infiltration of pathogenic Th17 cells, enhances expression of Th17‐related cytokines such as IL‐1β, IL‐6, and IL‐17, and exacerbates MRONJ development. This increased Th17 cell population and resultant cytokine secretion promote macrophage polarization toward a classically activated M1 pro‐inflammatory phenotype versus an alternatively activated M2 anti‐inflammatory phenotype.^(^ ^16^, ^17^, ^26^, ^105^ ^)^ Thus, a perpetual forward feedback loop is established that promotes inflammation and inhibits healing around areas of osteonecrosis.

#### Response of the oral mucosa

**Fig. 4:**
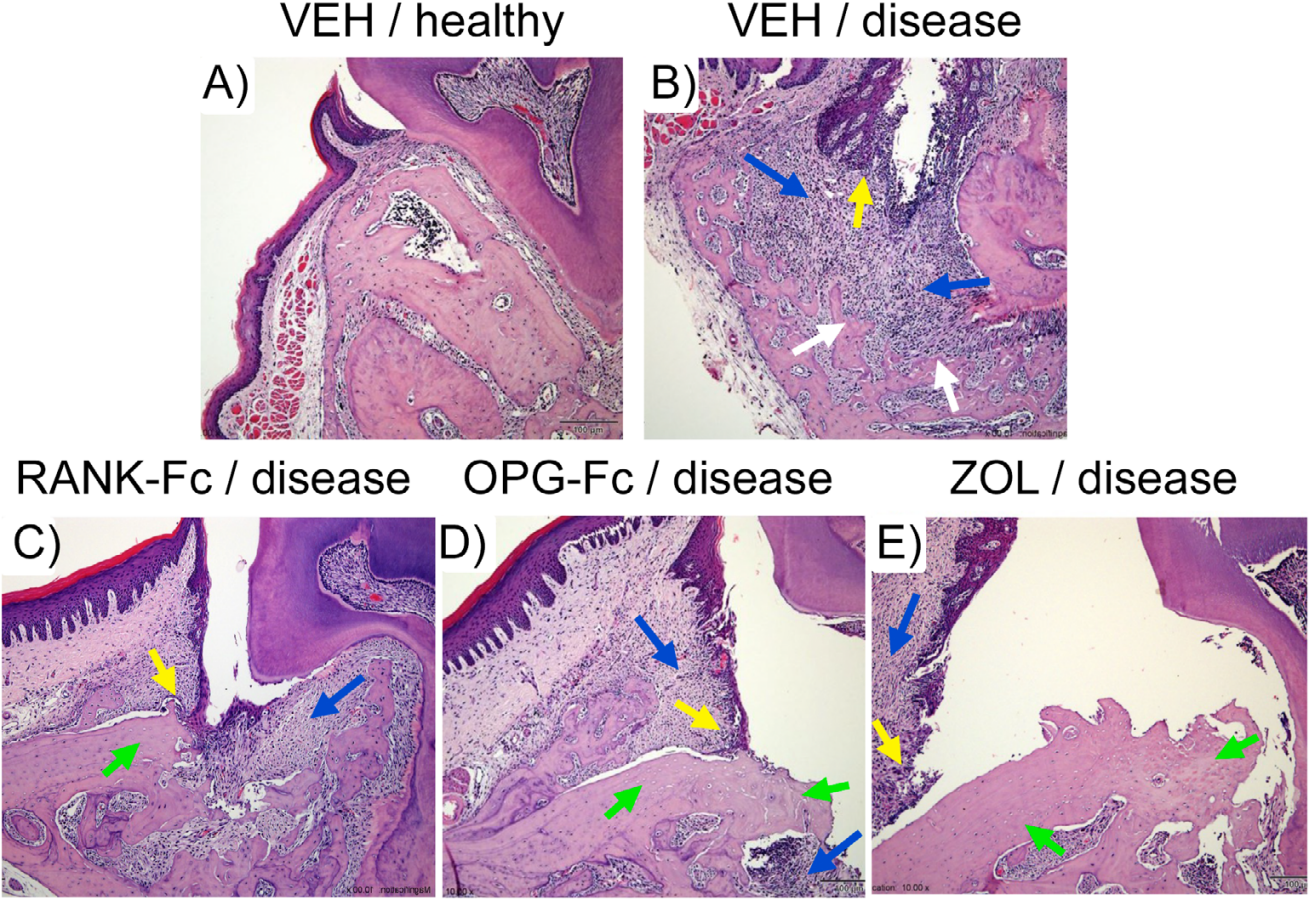
Progression of medication‐related osteonecrosis of the jaw (MRONJ) onset with developing bone exposure in mice treated with various antiresorptives. (*A*) Vehicle (VEH)‐treated healthy animal. (*B*) Vehicle‐treated animal with dental disease shows strong inflammatory infiltrate (blue arrow) and apical epithelial migration (yellow arrow). Alveolar bone is resorbed apically, away from the epithelium (white arrow). (*C*) RANK‐Fc (a denosumab surrogate)‐treated mouse with dental disease. Bone resorption is inhibited and necrotic bone is present at the alveolar crest (green arrow). Epithelial migration extends to the necrotic bone (yellow arrow). Inflammatory infiltrate is present (blue arrow). (*D*) OPG‐Fc (a denosumab surrogate)‐treated mouse with dental disease. Bone resorption is inhibited and necrotic bone is present at the alveolar crest (green arrows). Epithelium has migrated apically (yellow arrow) and reams the necrotic bone, which is exposed to the oral cavity. (*E*) Zoledronate (ZOL)‐treated mouse. Bone resorption is inhibited and necrotic bone is present at the alveolar crest (green arrows). Epithelium has migrated apically (yellow arrow) and demonstrates prominent reaming of the necrotic bone, resulting in extensive bone exposure.

In periodontal disease in the presence of antiresorptives, the junctional epithelium demonstrates apical proliferation. However, because of the inhibition of alveolar bone resorption, the distance between the migrating epithelium and the nonresorbed necrotic bone decreases.^(^ ^46^, ^47^, ^48^, ^101^ ^)^ Eventually, the migrating epithelium rims the necrotic bone, which becomes exposed to the oral cavity (Fig. 4).^(^ ^48^ ^)^ The molecular signals that lead to the epithelial migration are unknown. However, prominent expression of collagen type III and increased numbers of cells expressing high MMP‐9 and MMP‐13 levels or cells positive for a‐SMA, presumably myofibroblasts, are found apical to migrating epithelium and around sites of osteonecrosis.^(^ ^94^ ^)^ Interestingly, increased duration of periodontal disease augments the incidence of MRONJ,^(^ ^95^, ^105^ ^)^ while prevention or decrease of periodontal disease severity reduces MRONJ presence,^(^ ^106^ ^)^ demonstrating a time dependence between the two entities.

The close proximity of the alveolar bone to the infection nidus caused by the inhibition of bone resorption increases the probability of infection spreading to the necrotic bone.^(^ ^88^ ^)^ Indeed, bacterial presence is noted around osteonecrotic areas in rats treated with ZOL, but not in control animals, after extraction of teeth with periapical disease.^(^ ^90^ ^)^ In patients with clinical MRONJ lesions, a heavy biofilm formation is present on the necrotic bone surface.^(^ ^107^, ^108^, ^109^, ^110^ ^)^ The presence of infection most likely compromises the ability of soft and osseous tissues to heal because aggressive local wound care of exposed bone that reduces plaque accumulation and inflammation significantly improves overall disease resolution and decreases time to resolution.^(^ ^111^ ^)^

#### Tooth extraction

The setting of osteonecrosis and continued and augmented presence of inflammation result in clinical symptomatology that often requires extraction of the culprit teeth. Although frank bone exposure might not exist at the time, stage 0 MRONJ is often present and can escape clinical detection. Highly suppressed osteoclastic function and the resultant cellular and tissue changes from the preceding periodontal or periapical disease in combination with the added trauma of tooth extraction create a “perfect storm” that severely compromises healing of the extraction socket. Lack of woven bone formation, presence of empty socket, and poor healing of oral mucosa precipitate the setting of bone exposure and clinical MRONJ.^(^ ^90^, ^92^, ^93^, ^103^, ^112^, ^113^ ^)^ Both periodontal and periapical disease, as well as tooth extraction, increase turnover of the alveolar bone.^(^ ^114^, ^115^ ^)^ In these areas of localized increased turnover, higher accumulation of bisphosphonate deposition is expected.^(^ ^116^, ^117^, ^118^ ^)^

It is important to note that the great majority of published articles employing animal models of MRONJ utilize extraction of healthy teeth in the presence of antiresorptives. However, extraction of healthy teeth does not parallel the clinical experience. The vast majority of tooth extractions in adults is the sequel of dental caries, periodontal disease, periapical disease, or dental trauma.^(^ ^102^, ^119^, ^120^, ^121^, ^122^ ^)^ This is expected to be the case in patients on antiresorptives, where unnecessary extraction of healthy teeth would be contraindicated.

Select studies employing MRONJ animal models with extraction of healthy teeth in the presence of high doses of antiresorptives report abnormal healing of the extraction socket with presence of necrotic areas but clinically normal soft‐tissue healing.^(^ ^44^, ^104^, ^123^, ^124^, ^125^ ^)^ Importantly, adjunctive therapies, including steroids or chemotherapy, the presence of low vitamin D levels, or diabetes, in association with high doses of antiresorptives more predictably result in mucosal defects and exposure of the non‐healed extraction sockets.^(^ ^44^, ^104^, ^125^, ^126^ ^)^ These concomitant morbidities likely compromise mucosal and osseous healing and precipitate the occurrence of clinical disease.

**Fig. 5:**
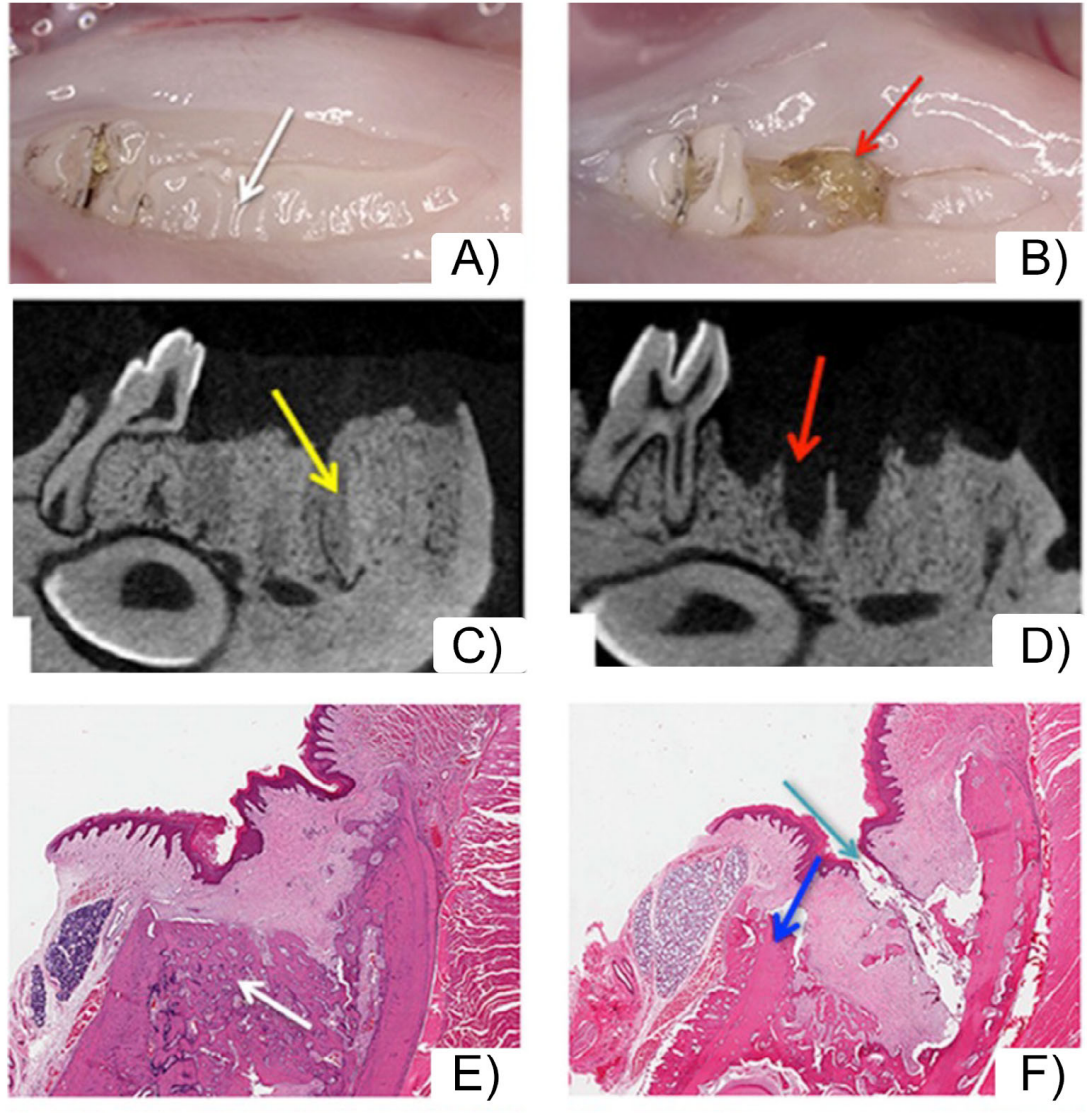
Extraction of healthy teeth or teeth with periapical disease in zoledronate (ZOL)‐treated animals. (*A*) Clinically extraction sockets of healthy teeth healed with normal mucosa (white arrows). (*B*) In contrast, extraction sockets of teeth with periapical disease showed areas of bone exposure (red arrows). (*C*) μCT assessment demonstrated extraction sockets of healthy teeth filled with woven bone (yellow arrow). Socket outlines are clearly demarcated. (*D*) Extraction sockets of teeth with periapical disease lack bone formation and appear empty, with socket outlines clearly defined (red arrows). (*E*) Histologic assessment demonstrates woven bone formation in the extraction socket of healthy teeth (white arrow). The margins of the extraction socket and the woven bone are clearly outlined. (*F*) In contrast, sockets of extracted teeth with periapical disease are void of bone formation, with the socket outlines visible and presence of osteonecrosis (blue arrows), debris, and bone exposure (aqua arrow). (Figure modified from Hadaya and colleagues^(^ ^90^ ^)^).

Interestingly, reports that directly compare extraction of healthy versus diseased teeth in the presence of antiresorptives report a higher incidence of MRONJ‐like features, including bone exposure, osteonecrosis, lack of socket healing, and persistence of inflammation after extraction of teeth with pre‐existing periapical or periodontal disease.^(^ ^90^, ^93^, ^94^, ^112^, ^113^ ^)^ Extraction of healthy teeth results in woven bone formation in the extraction socket, persistence of the original socket outlines due to inhibition of remodeling, and increased areas of necrotic bone, presumably because these necrotic areas cannot be removed in the absence of osteoclastic activity. However, the great majority of the extraction sockets heal with normal mucosa and the incidence of bone exposure is low (Fig. 5).^(^ ^90^ ^)^

## Proposed Model for the Pathogenesis of MRONJ

**Fig. 6:**
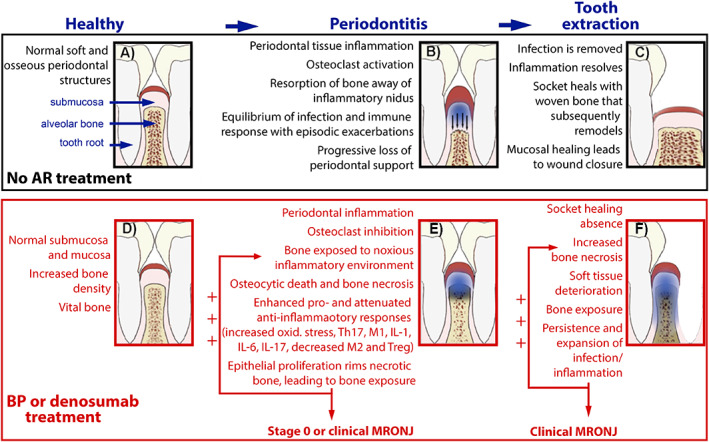
Proposed model for medication‐related osteonecrosis of the jaw (MRONJ) pathogenesis. AR = antiresorptive; BP = bisphosphonate.

Figure 6 depicts a proposed model for the pathogenesis of MRONJ. In the absence of antiresorptives and dental disease a healthy, functional equilibrium is achieved (Fig. 6*A*). Dental disease (periodontal disease is depicted as blue shaded area, Fig. 6*B*) leads to mucosal and submucosal inflammatory changes including osteoclast activation. The alveolar bone resorbs away of the inflammatory nidus (black arrows, Fig. 6*B*). A chronic inflammation is established with episodic exacerbations and progressive loss of periodontal support. Continued presence of dental disease eventually necessitates tooth extraction (Fig. 6*C*). Removal of the tooth eliminates the infectious stimulus, the inflammation resolves, and the extraction socket heals uneventfully and is covered by normal mucosa.

In the presence of antiresorptives and absence of dental disease, the healthy functional equilibrium is maintained. Increased trabecular density could be observed as a result of osteoclast inhibition; however, the alveolar bone remains vital (Fig. 6*D*). In the presence of dental disease (periodontal disease is depicted here), the inflammatory changes extend to the alveolar bone. The osteoclast inhibition impedes bone resorption and the alveolar bone is exposed to the inflammatory/infectious environment. Bone necrosis ensues (darkened alveolar bone, Fig. 6*E*). The presence of necrotic bone intensifies host responses with increased oxidative stress and a shift toward pro‐inflammatory cytokine secretion (IL‐17, IL‐1, IL‐6) and pro‐inflammatory immune cell (M1 macrophage polarization, increased Th17) presence, while anti‐inflammatory immune cell (Tregs and M2 macrophages) numbers decrease. Apical epithelial migration and rimming of the necrotic bone can result in bone exposure. The enhanced inflammatory responses and potential bone exposure further compound bone cell damage and cause osteonecrosis expansion. Thus, a perpetual positive feedback loop is established that augments tissue injury. Clinically, at the early stages, the patient presents with stage 0 MRONJ symptoms, while subsequent bone exposure can lead to clinical MRONJ.

Persistence of dental disease ultimately leads to tooth extraction. The cellular changes from the onset of osteonecrosis and the added trauma from the extraction induce significant insult to the alveolar tissues and compromise soft and osseous healing. The extraction socket fails to heal, the area of infection/inflammation (blue shaded area) and osteonecrosis (darkened alveolar bone) expand and clinical MRONJ is established (Fig. 6*F*). The exposed bone is colonized by bacteria and the inflammatory response is further enhanced. The same perpetual positive feedback loop is established and soft and osseous tissue changes, typical of MRONJ, progress.

Based on the above model, removal of dental disease at early stages should halt the disease progress, reduce the need of tooth extraction, and decrease the MRONJ incidence. Indeed, clinical studies have demonstrated that oral preventive measures, including diagnosis and treatment of dental disease, decrease the incidence of MRONJ in oncologic patients.^(^ ^127^, ^128^ ^)^

## Future Directions of Translational MRONJ Research

MRONJ remains a rare but serious complication of antiresorptive medications that often impedes proper control of the systemic disease. Furthermore, the fear of developing adverse effects such as MRONJ or atypical femoral fractures decreases treatment compliance for patients who are or are scheduled to be on antiresorptives and raises concerns about an imminent crisis in the management of osteoporosis.^(^ ^129^, ^130^, ^131^ ^)^

Reducing MRONJ incidence and improving treatment outcomes would not only benefit the patients with the disease but also all patients on antiresorptive medications. However, several areas of MRONJ pathogenesis, progression, and prognosis remain unknown and limit effective disease management. Although a relationship with dose and duration of antiresorptives and incidence of MRONJ has been shown, definition of exposure levels (cumulative dose load, ie, mg equivalents of an antiresorptive/years of exposure) has not been established. The mechanistic contribution of systemic comorbidities (smoking, diabetes, autoimmune diseases, malignancies) or medications (chemotherapies, corticosteroids, immune modulators, antiangiogenics, signaling inhibitors) and of local risk factors (surgical procedures, dental disease, anatomic factors, oral microbiome) need to be delineated in detail. Genetic determinants and prognostic biomarkers need to be validated such that individuals at higher risk to develop the disease or at subclinical stages before developing frank symptomatology are effectively identified. Usefulness of drug “holidays” in mitigating the risk of developing MRONJ or in reducing the severity of established disease, without compromising control of the systemic disease, need to be clarified. Effectiveness of nonoperative versus operative approaches for various MRONJ stages, antiresorptive regimens, and patient condition needs to be demonstrated, such that patients and providers can make evidence‐based informed decisions. The possible contribution of inherent differences of cellular responses to antiresorptives between the jaws versus other skeletal sites needs to be investigated further. Finally, discovery of targeted pharmacologic interventions to manipulate and support the mucosal, osseous, immune, and vascular healing response of the oral tissues and decrease or eliminate MRONJ burden would expand the armamentarium of approaches for MRONJ management.

The low incidence of MRONJ makes recruitment of large patient cohorts challenging to obtain and prospective studies difficult to design. Animal models that capture key clinical, radiographic, and histologic features of the disease offer important tools to assess key variables of disease establishment, progression, overall burden, and intervention effectiveness. Species that have been used in animal models of MRONJ include rat, mouse, rice rat, rabbit, dog, sheep, and pig.^(^ ^132^ ^)^ Although translational animal studies can provide valuable insights, several methodological challenges complicate data interpretation, comparison across studies, and conclusive relevance to the clinical reality. Such challenges include:

- Differences in the structure of soft and hard tissues between humans versus animals. This is particularly important for studies utilizing small rodents, where the cortical bone has a lamellar versus Haversian canal structure and does not undergo intracortical remodeling except for in response to interventions.^(^ ^133^, ^134^ ^)^ Potential implications of such structural and physiological differences in MRONJ pathophysiology are currently unclear.
- The need to utilize suprapharmacologic doses of antiresorptives in an effort to increase the incidence of MRONJ. Thus, the possibility of off‐target cellular and molecular effects that might not occur in the clinical setting should always be considered.
- Antiresorptive treatment regimens including dose, course of treatment, total cumulative dose, route of administration, presence of concomitant therapies (chemotherapy, anti‐angiogenic factors, immunomodulators, etc), duration of pretreatment before or of treatment after implementation of local instigating factors (ie, tooth extraction, experimental periodontal or periapical disease, implant placement, etc.) vary significantly among various animal studies.
- The uniform utilization of criteria that define the presence of MRONJ in animal models. Clinically or histologically present bone exposure is used in many but not all studies. Often the presence of histologically necrotic bone is the only parameter reported as the presence of MRONJ. The mere presence of histologically necrotic bone without evidence of exposure does not parallel the clinical diagnosis of the disease. Even stage 0 MRONJ that does not present bone exposure is characterized by nonspecific clinical and/or radiographic findings and not only by the presence of bone areas with empty osteocytic lacunae.
- The utilization of healthy teeth extractions as a local instigating factor in the majority of animal studies, that, as described earlier, does not parallel the clinical reality.
- The technically challenging surgical procedure of tooth extraction, particularly if the crown integrity has been compromised, that often results in root fragments remaining within the extraction sockets, often found in published reports. Such root fragments compromise proper socket healing in the absence or presence of antiresorptives and do not reflect the clinical setting. Animals, or at least extraction sockets, with radiographic or histologic evidence of root fragment presence should be excluded from data analyses.

Several of the above challenges are intimately related with the use of animal studies to explore human diseases, such as structural differences of osseous structures, differences in cortical bone remodeling physiology, or the need to administer suprapharmacologic antiresorptive doses. However, others can and should be addressed to create uniform and accepted models for translational studies. The field of MRONJ research would greatly benefit and advance from standardized antiresorptive treatment regimens; clearly established clinical, radiographic, and histologic criteria for disease presence, progression, severity, and resolution; clinically relevant and consistent across studies implementation of local instigating interventions to induce the disease; and rigorous and meticulous technical approaches to eliminate erroneous findings.

## Author Cotributions

ST: Conceptualization, creation of original draft, review and editing.

MRA: Conceptualization, creation of original draft, review and editing.

SLR: Conceptualization, creation of original draft, review and editing.

## Conflicts of Interest

The authors declare that they do not have any real or perceived conflicts with the content of the manuscript. SLR is a consultant for Amgen. MRA has an active MTA with Amgen and research grants from MBX Biosciences. Neither of these is for work related to the contents of the current article.

### Peer Review

The peer review history for this article is available at https://www.webofscience.com/api/gateway/wos/peer-review/10.1002/jbm4.10785.

## Data Availability

This is a review manuscript. No data were generated.
